# Weight Gain During SSRI Therapy: The Potential Role of the Gut Microbiota—A Narrative Review and Mechanistic Hypothesis

**DOI:** 10.3390/nu18182940

**Published:** 2026-09-08

**Authors:** Julia Śnieżek, Ewelina Polak-Szczybyło

**Affiliations:** 1 Student Scientific Club of Human Nutrition, Faculty of Health Sciences and Psychology, Collegium Medicum, University of Rzeszów, ul. Warzywna 1a, 35-959 Rzeszow, Poland; js128197@stud.ur.edu.pl; 2 Department of Dietetics, Faculty of Health Sciences and Psychology, Collegium Medicum, University of Rzeszów, ul. Warzywna 1a, 35-959 Rzeszow, Poland

**Keywords:** selective serotonin reuptake inhibitors, gut microbiota, antidepressant-associated weight gain, gut–brain axis, obesity, short-chain fatty acids, dietary interventions, psychopharmacomicrobiomics

## Abstract

**Introduction**: Selective serotonin reuptake inhibitors (SSRIs) remain the first-line pharmacological treatment for depressive disorders. However, long-term treatment is often accompanied by weight gain, which negatively impacts medication adherence and cardiometabolic health. Emerging evidence suggests that, in addition to their effects on the central nervous system (CNS), SSRIs may alter the composition and metabolic activity of the gut microbiome, potentially contributing to metabolic disorders. However, the mechanisms linking SSRI-induced microbial changes to weight regulation remain poorly understood. **Methods:** This narrative review summarizes the current evidence regarding the interactions between SSRIs, the gut microbiome, host metabolism, and weight regulation. Experimental, translational, and clinical studies were critically assessed, with particular emphasis on recent systematic reviews, meta-analyses, randomized controlled trials, and mechanistic studies. A conceptual mechanistic framework was developed based on the available evidence. **Results:** Experimental and clinical studies indicate that SSRIs can modify both the taxonomic composition and functional activity of the gut microbiota. These changes may influence the production of microbiota-derived metabolites, including short-chain fatty acids, bile acids, and tryptophan metabolites, thereby influencing gut barrier integrity, low-grade inflammation, gut hormone secretion, appetite regulation, and energy homeostasis. Current evidence also suggests that dietary interventions—including increased dietary fiber intake, adherence to a Mediterranean diet, and microbiota-targeted strategies using prebiotics, probiotics, synbiotics, and postbiotics—can beneficially modulate these pathways. However, no randomized, controlled trials have specifically assessed whether such interventions prevent SSRI-associated weight gain. **Conclusions:** Taken together, the available evidence raises the possibility that gut microbiota alterations may contribute to SSRI-associated weight gain, although this hypothesis has not yet been directly tested in studies evaluating the complete mechanistic pathway.

## 1. Introduction

Depressive disorders are among the most prevalent and debilitating diseases worldwide. It is estimated that approximately 4% of the global population is affected by depression, including 5.7% of adults (4.6% of men and 6.9% of women) and 5.9% of individuals aged 70 years and older [1]. A depressive episode is characterized by persistent low mood, sadness, feelings of emptiness or hopelessness, reduced self-esteem, and recurrent thoughts of death or suicide, with symptoms lasting for most of the day, nearly every day, for at least two weeks [1]. Pharmacological management of depressive and anxiety disorders is primarily based on selective serotonin reuptake inhibitors (SSRIs) and serotonin–norepinephrine reuptake inhibitors (SNRIs), which have remained the most commonly prescribed classes of psychiatric medications for several decades [2,3]. Among the most frequently prescribed SSRIs are sertraline, escitalopram, and fluoxetine. These agents effectively alleviate depressive and anxiety symptoms by inhibiting serotonin reuptake, thereby enhancing serotonergic neurotransmission [4]. SSRIs competitively inhibit the serotonin transporter (SERT), preventing serotonin reuptake into the presynaptic neuron and increasing its concentration within the synaptic cleft, ultimately potentiating serotonergic signaling [5]. SNRIs exert a similar mechanism of action but additionally inhibit the norepinephrine transporter (NET), resulting in increased synaptic concentrations of both serotonin and norepinephrine [6]. Despite their well-established efficacy, the long-term use of SSRIs and SNRIs is frequently limited by metabolic adverse effects, particularly weight gain, which may compromise treatment adherence and contribute to premature discontinuation of therapy [6,7,8,9]. Weight gain during antidepressant pharmacotherapy may become a clinically significant adverse effect; however, its frequency and magnitude depend on treatment duration, baseline body weight, patient characteristics, and the specific drug used. In the population-based study by Gafoor et al., clinically significant weight gain was defined as an increase in body weight of ≥5%. The incidence of such weight gain was 11.2 per 100 person-years among individuals using antidepressants, compared with 8.1 per 100 person-years among non-users. The risk remained elevated for at least six years of follow-up, and the analysis accounted for factors including age, sex, baseline BMI, comorbidities, smoking status, concomitant medication use, and dietary advice [7]. Furthermore, SSRIs constitute a heterogeneous group in terms of their effects on body weight. A recent comparative study involving 183,118 patients showed that, after six months of treatment, weight gain was greater with escitalopram and paroxetine than with sertraline, by 0.41 and 0.37 kg, respectively. Citalopram was associated with a slightly greater weight gain of 0.12 kg, whereas no significant difference was observed between fluoxetine and sertraline. Escitalopram and paroxetine were also associated with a 10–15% higher risk of gaining ≥5% of baseline body weight [8]. Data on fluvoxamine are considerably more limited; therefore, its effects on body weight remain less well characterized. Accordingly, the assessment of SSRI-associated weight gain should consider treatment duration, patient-related factors, and the specific active substance used. Psychological distress associated with changes in body weight and metabolic disturbances is frequently reported as one of the leading reasons for premature discontinuation of antidepressant therapy, thereby increasing the risk of depressive relapse [10]. Growing evidence indicates that antidepressants, although primarily developed to modulate neurotransmission within the central nervous system (CNS), also exert systemic effects, including alterations in the composition and metabolic activity of the gut microbiota [11]. These microbiota-related effects appear to be bidirectional. On the one hand, antidepressant-induced modulation of the gut microbiota may contribute to improved therapeutic efficacy. On the other hand, accumulating evidence suggests that drug-induced microbial alterations may increase susceptibility to adverse metabolic effects and contribute to interindividual variability in treatment response. Because bacterial enzymes participate in drug biotransformation, the gut microbiota may influence drug metabolism, bioavailability, efficacy, and the occurrence of adverse effects [7,11].

In recent years, increasing attention has been devoted to the emerging field of psychopharmacomicrobiomics, which describes the bidirectional interactions between psychotropic medications and the gut microbiota [12]. The impact of pharmacotherapy on gut microbial composition has been well documented [13]. It is estimated that approximately 25% of all drugs used in humans inhibit the growth of at least one gut bacterial strain, with psychotropic medications representing a substantial proportion of these compounds. Consequently, drug-induced alterations in the gut microbiota may significantly influence treatment efficacy, tolerability, and overall therapeutic outcomes by either attenuating or enhancing drug effects [12]. While the gut microbiota can influence the metabolism, bioavailability, and therapeutic efficacy of psychotropic drugs, these medications can, in turn, reshape the composition and functional capacity of the gut microbiome. Current evidence suggests that antidepressant treatment does not substantially alter the overall bacterial load of the gut microbiota compared with untreated individuals [14]. However, numerous antidepressants, antipsychotics, and mood stabilizers exhibit antimicrobial activity, resulting in selective changes in microbial composition. Such alterations may represent one of the mechanisms underlying differences in treatment efficacy, adverse effects, and interindividual variability in clinical response [15].

Although increasing evidence suggests that SSRIs can alter the composition and function of the gut microbiota, it remains unclear whether these changes contribute to SSRI-associated weight gain. Moreover, no comprehensive review has integrated the available evidence into a unified mechanistic framework linking SSRI-induced microbial alterations with metabolic outcomes. Therefore, the aim of this narrative review was to critically summarize current evidence regarding the interactions between SSRI therapy, the gut microbiota, and body weight regulation, and to propose a mechanistic framework integrating the available experimental and clinical findings.

## 2. Methods

This article was designed as a structured narrative review. The narrative approach was selected because the research question spans heterogeneous clinical, preclinical, microbiological, metabolic, and mechanistic evidence that could not be appropriately synthesized using a single narrowly defined framework. Nevertheless, structured database searching, predefined eligibility criteria, and documented study-selection procedures were applied to improve the transparency and reproducibility of the review. The manuscript was additionally evaluated using the Scale for the Assessment of Narrative Review Articles (SANRA)—Supplementary Table S1.

### 2.1. Information Sources and Search Strategy

The literature search was conducted in PubMed/MEDLINE, Scopus, and Web of Science and covered English-language publications issued between 1 January 2011 and 27 August 2026. The searches covered three predefined evidence domains: (1) associations between SSRI treatment and the composition or function of the gut microbiota; (2) associations between SSRI treatment and body weight or metabolic outcomes; and (3) microbiota-related mechanisms potentially linking SSRI treatment with body weight regulation. A supplementary search addressed dietary and microbiota-targeted interventions relevant to these mechanisms.

Four thematic searches were performed. The first combined SSRI-related terms (selective serotonin reuptake inhibitor, SSRI, antidepressant, sertraline, escitalopram, citalopram, fluoxetine, fluvoxamine, and paroxetine) with gut microbiota-related terms (gut microbiota, gut microbiome, intestinal microbiota, intestinal microbiome, dysbiosis, gut–brain axis, and psychopharmacomicrobiomics). The second combined the SSRI-related terms with terms concerning body weight and metabolic outcomes (weight gain, body weight, obesity, adiposity, appetite, satiety, energy balance, energy homeostasis, insulin resistance, and metabolic adverse effects). The third combined gut microbiota-related terms with metabolic and mechanistic terms (short-chain fatty acids, SCFA, acetate, propionate, butyrate, intestinal permeability, intestinal barrier, lipopolysaccharide, LPS, metabolic endotoxemia, inflammation, low-grade inflammation, tryptophan metabolism, kynurenine, serotonin, bile acids, GLP-1, and PYY). The fourth search addressed potential dietary and microbiota-targeted interventions and included the terms: dietary fiber, Mediterranean diet, prebiotic, probiotic, synbiotic, postbiotic, psychobiotic, fecal microbiota transplantation, and FMT, combined with SSRI-, depression-, microbiota-, and body-weight-related terms. Terms within each conceptual group were combined using OR, whereas the conceptual groups were combined using AND.

Only publications in English were searched.

### 2.2. Eligibility Criteria

Eligible publications included: (1) clinical studies examining body weight, appetite, body composition, metabolic outcomes, or gut microbiota characteristics in individuals receiving SSRIs; (2) controlled animal or in vitro studies investigating the effects of SSRIs on gut microorganisms or microbiota-related metabolic pathways; (3) systematic reviews, scoping reviews, and meta-analyses relevant to SSRI-associated weight change, microbiota–host metabolic interactions, or microbiota-targeted interventions; (4) narrative and mechanistic reviews relevant to the conceptual framework; and (5) mechanistic studies directly addressing the proposed pathways, including intestinal permeability, microbial metabolites, inflammation, tryptophan metabolism, and enteroendocrine signaling. Studies concerning antidepressants other than SSRIs were eligible only when SSRI-specific findings were reported separately.

Publications were excluded if they: (1) did not address SSRIs, the gut microbiota, body weight regulation, or a directly relevant mechanistic pathway; (2) did not allow SSRI-specific findings to be distinguished from those concerning other psychotropic medications; (3) were conference abstracts, protocols, editorials, letters without original data, dissertations, or other non-peer-reviewed publications; or (4) had no full text available. Only articles published in English were included. Studies of dietary or microbiota-targeted interventions conducted outside the context of SSRI treatment were used solely to discuss indirect mechanistic evidence and were not considered evidence that these interventions prevent SSRI-associated weight gain.

The final evidence synthesis included 91 peer-reviewed publications: 38 original studies, comprising 16 human studies, 12 preclinical or in vivo studies, and 9 in vitro or basic experimental studies; 16 systematic or scoping reviews and meta-analyses; and 37 narrative, mechanistic, or expert reviews. Three additional sources were cited solely to provide contextual background and were not included in the evidence synthesis.

### 2.3. Study Selection

The database searches and initial screening of titles and abstracts were performed by the first author. Publications considered potentially relevant were subsequently assessed in full text according to the predefined eligibility criteria. The second author participated in the full-text eligibility assessment and verified the final selection of sources. Any uncertainties regarding eligibility were resolved through discussion between the authors. The reference lists of relevant reviews and original studies were also screened to identify additional eligible publications. The final evidence synthesis included 91 peer-reviewed publications, while three additional sources were used solely to provide contextual information.

### 2.4. Evidence Appraisal and Synthesis

Because of substantial heterogeneity in study designs, populations, exposures, and outcomes, no quantitative synthesis was performed. The evidentiary weight of each publication was considered according to predefined criteria: study design, sample size, presence of an appropriate comparator, duration of follow-up, validity of exposure and outcome measurements, control of relevant confounding factors, directness to the review question, and consistency with other clinical and experimental findings. Evidence obtained directly from patients receiving SSRIs was distinguished from indirect evidence derived from other populations or experimental models. Studies were not excluded solely on the basis of this appraisal, rather, the assessment was used to calibrate the strength of the conclusions.

### 2.5. Distinction from a Systematic Review

This review should not be considered a systematic review. No protocol was prospectively registered, the review was not restricted to a single narrowly defined clinical question, and no formal meta-analysis or certainty-of-evidence assessment was undertaken. Its purpose was to integrate heterogeneous evidence and develop a hypothesis-generating mechanistic framework. The structured search and selection procedures were introduced to improve transparency and reproducibility but do not imply exhaustive systematic-review methodology.

## 3. SSRIs as Modulators of the Gut Microbiota

SSRIs are currently among the most commonly prescribed antidepressants in clinical practice. Their widespread use is primarily attributable to their well-documented efficacy, more favorable safety profile compared with tricyclic antidepressants and monoamine oxidase inhibitors, and relatively good tolerability. SSRIs are considered first-line pharmacological treatments for major depressive disorder (MDD), anxiety disorders, obsessive compulsive disorder, post-traumatic stress disorder, and several other psychiatric conditions in which alterations in serotonergic neurotransmission play an important pathophysiological role [16]. The primary mechanism of action of SSRIs involves the selective inhibition of the serotonin transporter (SERT), resulting in increased serotonin availability within the synaptic cleft and enhanced serotonergic neurotransmission in the central nervous system. However, growing evidence indicates that the biological effects of SSRIs extend beyond the brain. This is partly attributable to the widespread distribution of SERT and serotonin receptors in peripheral tissues, particularly in the gastrointestinal tract, which contains approximately 90–95% of the body’s total serotonin pool. In the gut, serotonin regulates numerous physiological processes, including gastrointestinal motility, secretion, immune responses, and communication along the gut–brain axis [17,18].

In recent years, increasing attention has been paid to the non-neuronal effects of SSRIs, including their potential interactions with the gut microbial ecosystem. Experimental studies indicate that some psychotropic drugs possess antimicrobial properties, including antibacterial and antifungal activity and, in certain cases, antiviral effects [19]. Although the available evidence is derived primarily from in vitro studies and, to a lesser extent, from animal and clinical studies, it suggests that psychotropic drugs may directly affect the gut microbial ecosystem. Proposed mechanisms include disruption of microbial cell membrane integrity, inhibition of bacterial efflux pumps, interference with microbial metabolic pathways and energy production, modulation of virulence factor expression, and inhibition of biofilm formation. Furthermore, psychotropic drugs may enhance the efficacy of conventional antibiotics by attenuating bacterial resistance mechanisms, representing a potential direction for future therapeutic strategies [19,20]. Direct antimicrobial activity may therefore constitute one of the mechanisms through which SSRIs modify the gut microbial environment, although the relevance of these effects to the composition and function of the human gut microbiota remains to be established.

Animal studies have consistently demonstrated that antidepressant treatment alters the overall composition of the gut microbiota compared with that of untreated animals. By contrast, findings from human studies are less consistent, potentially because of substantial interindividual variability associated with diet, lifestyle, environmental exposures, and other host-related factors that influence microbiota composition [14]. Differences between preclinical and clinical findings should therefore be considered when interpreting the potential biological consequences of SSRI exposure, as changes observed under controlled experimental conditions may not be directly translatable to patients receiving antidepressant treatment.

Recent clinical studies have begun to identify specific microbial taxa associated with antidepressant use. Lin et al. reported reduced abundances of several bacterial genera, including *Turicibacter*, *Barnesiella*, *Akkermansia*, *Dialister*, *Romboutsia*, and *Fusicatenibacter*, as well as members of the Lachnospiraceae ND3007 group, in patients receiving SSRIs or SNRIs compared with untreated individuals (*p* < 0.05). Notably, the abundance of *Turicibacter* was inversely correlated with the severity of depressive symptoms (r = −0.43; *p* < 0.05), suggesting a potential association between gut microbiota composition and clinical outcomes [21]. Observational studies have also reported a reduced abundance of *Faecalibacterium* in individuals receiving antidepressant treatment [22]. This genus includes some of the major butyrate-producing bacteria. Butyrate is a short-chain fatty acid (SCFA) with well-established anti-inflammatory properties and an important role in maintaining intestinal barrier integrity. Similarly, bacteria belonging to the genus *Bifidobacterium* contribute to intestinal homeostasis through SCFA production, regulation of immune responses, reinforcement of epithelial barrier function, modulation of tryptophan metabolism, and maintenance of normal gut–brain axis communication. However, the available evidence regarding the effects of antidepressants on *Bifidobacterium* abundance remains inconsistent, with findings varying according to the antidepressant used and the characteristics of the study population [23].

The available evidence suggests that SSRIs may influence the composition and function of the gut microbiota through both indirect host-mediated mechanisms and direct antimicrobial activity. At the same time, the existing data are heterogeneous with respect to the specific SSRI investigated, study design, and study population, with preclinical findings generally being more consistent than those obtained in humans. Importantly, evidence supporting a potential role of the microbiota in antidepressant efficacy should be clearly distinguished from evidence concerning its possible contribution to SSRI-associated weight gain. Although the former hypothesis is supported by a growing body of preclinical and clinical research, evidence directly linking SSRI-induced microbiota alterations to subsequent weight gain remains limited and is derived primarily from preclinical studies. Accordingly, the following subsections discuss individual SSRIs separately, taking into account the type of evidence (in vitro, animal, or clinical) and, where data are available, distinguishing findings related to antidepressant efficacy from those potentially relevant to SSRI-associated weight gain.

### 3.1. Sertraline

Sertraline is one of the most widely prescribed SSRI and the antidepressant whose antimicrobial properties have been the most extensively investigated [19]. Interest in its antimicrobial activity emerged following in vitro studies demonstrating growth inhibition of selected bacterial and fungal species at concentrations unrelated to its primary pharmacological action within the CNS [22]. As orally administered SSRIs first come into contact with the gut microbiota in the gastrointestinal tract, where relatively high luminal drug concentrations are reached before systemic absorption, increasing attention has been directed toward their potential to directly modulate intestinal microorganisms independently of their classical serotonergic effects. Indeed, several SSRIs have been shown to possess antimicrobial properties capable of altering both the composition and functional activity of the gut microbiota [19].

One of the first studies providing direct evidence that SSRIs can modulate the gut microbiota was conducted by McVey Neufeld et al. Chronic administration of sertraline in mice reduced gut microbial α-diversity and selectively altered the abundance of specific bacterial taxa while largely preserving the overall microbial community structure. Notably, these microbial changes persisted following vagotomy, indicating that sertraline-induced modulation of the gut microbiota occurs independently of vagal signaling. In contrast, the antidepressant effects of sertraline required an intact vagus nerve, suggesting that SSRIs may exert two parallel biological actions: direct modulation of the gut microbiota and regulation of the gut–brain axis through vagal pathways [24]. These findings provided some of the earliest experimental evidence that the gut microbiota may represent a direct target of antidepressant therapy, independently of CNS activity. Importantly, however, this study was conducted in an animal model and therefore provides mechanistic rather than clinical evidence. Moreover, although it supports a potential role of microbiota-related mechanisms in antidepressant action, it does not demonstrate that microbiota alterations contribute to SSRI-associated weight gain.

Beyond its effects on the gut microbial community, sertraline has demonstrated broad direct antimicrobial activity, although the available evidence is predominantly derived from in vitro studies. Sertraline has been reported to inhibit microbial proliferation, reduce adhesion to biological surfaces, and impair biofilm formation [19,20,25,26]. Antimicrobial effects have been described against a wide range of clinically relevant microorganisms, including Gram-positive bacteria, particularly *Staphylococcus aureus* and *Enterococcus faecalis*, Gram-negative bacteria such as *Escherichia coli*, *Pseudomonas aeruginosa*, and *Acinetobacter baumannii*, as well as fungi belonging to the genus *Candida*, especially *Candida albicans* [20,25]. Ahmed et al. demonstrated that sertraline significantly reduced the biomass and metabolic activity of pre-established *C. albicans* biofilms while simultaneously decreasing the expression of genes involved in adhesion and biofilm maturation, indicating that its activity extends beyond inhibition of planktonic growth to disruption of mature biofilm architecture [25]. Similar inhibitory effects have also been observed in preformed biofilms [27].

An increasing number of in vitro studies further suggest that sertraline may potentiate the activity of conventional antimicrobial agents. In vitro experiments have demonstrated synergistic interactions between sertraline and antifungal drugs, including fluconazole and amphotericin B, against both planktonic cells and fungal biofilms. These effects are thought to result from disruption of membrane integrity, altered membrane homeostasis, and enhanced intracellular accumulation of antifungal agents through modulation of membrane transport processes [28,29]. The precise mechanisms underlying the antimicrobial activity of sertraline remain incompletely understood and are likely multifactorial. Current evidence suggests that sertraline disrupts membrane homeostasis, interferes with microbial metabolic pathways, reduces adaptive capacity, and inhibits biofilm formation. Unlike conventional antibiotics, which typically target a single cellular structure or metabolic pathway, sertraline appears to affect multiple microbial processes simultaneously. Such pleiotropic activity may be particularly relevant in the context of the growing global burden of antimicrobial resistance, as targeting several cellular pathways simultaneously could reduce the likelihood of rapid resistance development [19,30].

A further consideration concerns the broader ecological consequences of SSRI exposure, which may be relevant to the long-term effects of these drugs on microbial ecosystems. Preclinical studies indicate that, in addition to its direct antimicrobial activity, sertraline may influence bacterial physiology and responses to antibiotics through modulation of efflux pump activity, increased oxidative stress, alterations in membrane integrity, and promotion of persister-cell formation [31,32,33]. Sertraline has also been shown to increase the frequency of horizontal transfer of antibiotic-resistance genes by promoting bacterial transformation [34]. However, these observations have been obtained predominantly from in vitro studies and bacterial models and cannot be directly extrapolated to clinical conditions. They nevertheless indicate that prolonged SSRI exposure may exert selective pressure on microorganisms and potentially modify functional properties of the intestinal microbial ecosystem. From the perspective of the present review, these findings are relevant primarily as a potential mechanistic background rather than as direct evidence of weight regulation. Alterations in microbial functionality and ecosystem stability could theoretically influence metabolic pathways involved in appetite regulation, energy metabolism, and body weight; however, the available studies do not establish such a causal relationship.

Overall, available evidence indicates that sertraline can both directly interact with microorganisms and alter the gut microbial ecosystem. The evidence encompasses direct antimicrobial activity, inhibition of biofilm formation, modulation of microbial membrane and metabolic processes, and, in animal models, alterations in gut microbial diversity and composition. However, these findings derive predominantly from in vitro experiments and animal studies, whereas evidence from human populations remains limited. Consequently, the physiological and clinical significance of sertraline-induced microbial alterations remains uncertain. Importantly, the available evidence should be interpreted according to the hypothesis it addresses: experimental findings involving vagal signaling and antidepressant effects provide preliminary support for the potential involvement of the gut microbiota in antidepressant action [24], whereas the proposed link between microbiota alterations and SSRI-associated weight gain remains largely hypothetical and is not directly demonstrated by the studies summarized above. Further clinical and translational studies are therefore required to determine whether sertraline-induced changes in microbial composition or function contribute to alterations in appetite, energy metabolism, or body weight.

To facilitate comparison across experimental models and to distinguish evidence related to antidepressant efficacy from that concerning potential metabolic effects, the available findings on sertraline-associated changes in gut microbiota composition and function are summarized in Table 1.

### 3.2. Escitalopram

Escitalopram is another widely prescribed SSRI, and accumulating evidence suggests that its therapeutic efficacy may be partly mediated through its effects on the gut microbiota. Reported alterations involve both the taxonomic composition of the gut microbial community and its metabolic activity, potentially contributing to modulation of the gut–brain axis and influencing the clinical response to antidepressant treatment [35]. Importantly, the available evidence primarily supports a potential role of the gut microbiota in antidepressant efficacy, whereas its contribution to SSRI-associated weight gain remains insufficiently investigated.

One of the first clinical studies investigating the effects of SSRI therapy on the gut microbiota was the prospective study conducted by Shen et al., which included treatment-naïve patients experiencing their first episode of MDD [36]. Compared with healthy controls, patients with MDD exhibited significant gut microbial dysbiosis prior to treatment, characterized by an increased Firmicutes-to-Bacteroidetes ratio and altered α- and β-diversity. Following 4–6 weeks of escitalopram therapy, a partial normalization of the gut microbiota was observed, including a reduction in the Firmicutes-to-Bacteroidetes ratio, an increased relative abundance of *Bacteroides*, and partial restoration of microbial diversity. These findings suggest that successful antidepressant therapy may partially reverse depression-associated microbial alterations. However, functional analysis of the gut microbiome revealed that metabolic differences between treated patients and healthy controls persisted despite clinical improvement, indicating that restoration of microbial function may occur more slowly than symptom resolution [36]. Although this study provided some of the earliest clinical evidence linking escitalopram treatment with changes in gut microbiota composition, its relatively small sample size, lack of randomization, and short follow-up period warrant cautious interpretation, and the findings should be considered hypothesis-generating rather than conclusive [36].

Further insight into the potential relationship between the gut microbiota and escitalopram response was provided by the prospective study of Wang et al. (2023), which included 110 patients with MDD treated with escitalopram for 12 weeks [37]. The authors employed a multi-omics approach integrating shotgun metagenomic sequencing of the gut microbiome with plasma metabolomic profiling, enabling simultaneous assessment of microbial composition and functional metabolic consequences. Baseline differences in both gut microbial composition and metabolic profiles were observed between responders and non-responders, suggesting that the initial characteristics of the gut microbiome may influence antidepressant efficacy. The most pronounced differences involved microbial pathways related to tryptophan metabolism and indole biosynthesis, both of which play important roles in gut–brain axis signaling. Notably, the abundance of specific bacterial taxa was positively associated with circulating concentrations of indole-3-propionic acid (IPA), a microbiota-derived tryptophan metabolite with anti-inflammatory, antioxidant, and neuroprotective properties. Higher IPA concentrations were associated with a more favorable therapeutic response, suggesting that microbiota-derived metabolites may contribute to the mechanisms underlying escitalopram efficacy. Collectively, these findings indicate that the functional metabolic activity of the gut microbiota may be a more important determinant of treatment response than taxonomic composition alone [37].

These clinical observations are further supported by experimental studies in animal models. In two independent studies, escitalopram treatment was associated with partial restoration of intestinal eubiosis accompanied by metabolic remodeling of the host [38,39]. Treatment response correlated not only with alterations in microbial composition but, more importantly, with changes in metabolic pathways involved in phospholipid and sphingolipid metabolism. Together with the clinical findings, these results suggest that the functional capacity of the gut microbiota may play a greater role in antidepressant efficacy than changes in microbial taxonomy alone [38,39].

Overall, the available clinical and preclinical evidence indicates that escitalopram is associated with changes in both the composition and functional activity of the gut microbiota, with the strongest evidence relating to its potential role in antidepressant treatment response. In particular, alterations in microbial metabolic pathways involved in tryptophan metabolism, lipid metabolism, and the production of signaling metabolites participating in gut–brain axis communication may be more relevant to treatment efficacy than taxonomic alterations alone [37,38,39]. Nevertheless, current evidence does not allow a definitive conclusion as to whether these microbiota-related changes represent a direct pharmacological effect of escitalopram or a secondary consequence of clinical improvement. This represents an important limitation, as improvement in depressive symptoms may itself modify several factors known to influence the gut microbiota and body weight, including appetite and dietary composition, physical activity, sleep, inflammatory status, and metabolic parameters. Consequently, the partial normalization of the gut microbiota observed during treatment cannot currently be attributed specifically to a direct effect of escitalopram [36].

Overall, the available clinical and preclinical evidence indicates that escitalopram is associated with changes in both the composition and functional activity of the gut microbiota, with current evidence supporting a potential role of microbiota-related metabolic pathways—particularly those involved in tryptophan and lipid metabolism and the production of gut–brain axis signaling metabolites—in antidepressant treatment response [37,38,39]. However, it remains unclear whether these changes result directly from escitalopram treatment or occur secondarily to clinical improvement, which may itself influence factors such as appetite, dietary composition, physical activity, sleep, inflammatory status, and metabolic parameters [36]. Although these findings provide a biological rationale for considering the gut microbiota as a potential mediator of metabolic effects during SSRI treatment, the available studies have not directly investigated microbiota-mediated weight gain. Thus, current evidence supports a potential contribution of the gut microbiota to escitalopram efficacy but does not yet establish its role in SSRI-associated weight gain, highlighting the need for well-designed translational and prospective clinical studies integrating metagenomic and metabolomic approaches while controlling for relevant confounding factors.

The available clinical and preclinical evidence regarding escitalopram-associated alterations in the gut microbiota and their potential relationship with antidepressant treatment response is summarized in Table 2.

### 3.3. Fluoxetine

Similar to sertraline and escitalopram, fluoxetine has been shown to modulate the gut microbiota. Current evidence indicates that fluoxetine influences both the taxonomic composition and the functional activity of the gut microbial community. Reported changes include increased microbial diversity, attenuation of stress-induced dysbiosis, and partial restoration of metabolic pathways involved in gut–brain axis communication. These microbiota-related effects have been proposed as one of the mechanisms contributing to the antidepressant efficacy of fluoxetine [40].

Evidence that fluoxetine may directly influence intestinal bacteria comes from studies examining its effects on bacterial growth, metabolism, and colonization. Fung et al. investigated fluoxetine–microbiota interactions using mouse models and the commensal bacterium *Turicibacter sanguinis* [41]. The authors demonstrated that this species possesses a transporter structurally and functionally similar to the mammalian SERT and actively imports serotonin from the intestinal lumen. Fluoxetine inhibited this process, altering the expression of genes involved in membrane transport, metabolism, and sporulation and ultimately reducing the bacterium’s capacity to colonize the gastrointestinal tract. Consistent with these findings, mice receiving fluoxetine exhibited a significant reduction in the abundance of *T. sanguinis*. These results indicate that fluoxetine may directly modulate gut microbial composition by affecting the growth, metabolic activity, and colonization capacity of specific bacterial species [41]. Additional evidence for direct effects of fluoxetine on the gut microbiota was provided by Lyte et al. (2019), who investigated healthy mice without pre-existing microbiota disturbances [42]. Following 29 days of treatment, fluoxetine induced selective remodeling of the gut microbiota, including reduced abundances of *Lactobacillus* johnsonii and members of the Bacteroidales S24-7 group, together with increased abundances of *Anaerotruncus* and *Lachnoclostridium*. These alterations were accompanied by significant differences in body weight, suggesting that fluoxetine-induced changes in gut microbial composition may contribute to metabolic outcomes [42].

Experimental studies in models of stress and depression further support an association between fluoxetine treatment, restoration of gut microbial homeostasis, and antidepressant effects. Sun et al. investigated fluoxetine in a mouse model of chronic unpredictable mild stress (CUMS), in which chronic stress induced marked gut microbial dysbiosis characterized by reduced microbial diversity, simplified microbial interaction networks, and increased abundance of opportunistic bacteria, including members of the genera *Escherichia*/*Shigella*, *Enterococcus*, *Aerococcus*, and *Vagococcus* [43]. Fluoxetine treatment partially restored microbial diversity, reduced the abundance of opportunistic bacteria, and re-established a more balanced microbial community. Importantly, the degree of microbiota normalization positively correlated with improvements in depressive- and anxiety-like behaviors, suggesting that the antidepressant effects of fluoxetine may be partly mediated through restoration of gut microbial homeostasis and modulation of the gut–brain axis [43]. Similar findings were reported by Zhang et al. using a rat model of CUMS [40]. Chronic stress disrupted both the composition and functional capacity of the gut microbiota, whereas fluoxetine treatment induced substantial remodeling of the microbial community, reflected by significant changes in β-diversity and partial reversal of stress-induced dysbiosis. Fluoxetine also influenced multiple metabolic pathways involved in amino acid, carbohydrate, and lipid metabolism, vitamin biosynthesis, and the production of microbiota-derived metabolites. These findings suggest that restoration of microbial metabolic activity, rather than taxonomic composition alone, may represent a key determinant of the antidepressant efficacy of fluoxetine [40].

More direct evidence that the gut microbiota may actively mediate the antidepressant effects of fluoxetine was provided by Lee et al. in an LPS-induced mouse model of depression [44]. Fluoxetine treatment increased the abundance of *Lactobacillus*, and the magnitude of this increase positively correlated with improvements in depressive- and anxiety-like behaviors. Depletion of the gut microbiota using broad-spectrum antibiotics markedly attenuated the antidepressant efficacy of fluoxetine, whereas administration of *Limosilactobacillus reuteri* and *Ligilactobacillus murinus*, whose abundance increased during fluoxetine treatment, partially reproduced the antidepressant effects even in the absence of the drug. Furthermore, vagotomy abolished both the therapeutic effects of fluoxetine and the beneficial effects of bacterial supplementation, highlighting the essential role of vagal signaling in gut–brain communication. These findings provide experimental support for the hypothesis that specific gut bacterial species may act as active mediators of the antidepressant response to fluoxetine [44].

Fluoxetine may also influence the gut microbiota in ways that are relevant to metabolic outcomes. In healthy mice without pre-existing microbiota disturbances, Lyte et al. observed selective changes in bacterial taxa considered important for host metabolic homeostasis, together with increases in genera commonly associated with dysbiosis [42]. These microbial alterations were accompanied by significant differences in body weight, providing preliminary evidence that fluoxetine-induced microbiota remodeling may be associated with metabolic outcomes [42]. However, these findings do not establish that microbiota alterations mediate SSRI-associated weight gain and should therefore be distinguished from the evidence supporting microbiota involvement in antidepressant efficacy.

Another aspect of fluoxetine–microbiota interactions concerns antimicrobial activity and bacterial resistance. Ty et al. demonstrated that prolonged exposure of bacteria to sublethal concentrations of antidepressants, including fluoxetine, induced the expression of genes encoding multidrug efflux pumps, particularly those belonging to the resistance–nodulation–division (RND) family, including the AcrAB–TolC system [45]. Bacterial strains selected under SSRI exposure exhibited reduced susceptibility not only to antidepressants but also to multiple classes of antibiotics, indicating the development of cross-resistance. The involvement of efflux pumps was confirmed by the use of efflux pump inhibitors, which partially restored bacterial susceptibility to both antibiotics and antidepressants. These findings suggest that activation and selection of efflux-mediated resistance represent one of the principal mechanisms through which SSRIs may contribute to multidrug resistance, highlighting efflux pump inhibition as a potential strategy to mitigate this phenomenon [45].

Collectively, the available evidence indicates that fluoxetine exerts multifaceted effects on the gut microbiota, involving both direct interactions with specific bacterial species and broader changes in microbial composition and metabolic activity. Preclinical studies further suggest that microbiota remodeling, particularly changes in microbial metabolic pathways and microbiota-derived metabolites, may contribute to the antidepressant effects of fluoxetine, with experimental depletion and bacterial supplementation studies providing the strongest evidence for a potential mediating role of the microbiota [40,43,44]. At the same time, evidence linking fluoxetine-induced microbiota alterations to metabolic outcomes, including body weight, remains limited and does not establish microbiota-mediated SSRI-associated weight gain [42]. Overall, although the evidence is predominantly preclinical, it supports the concept that the gut microbiota may represent both a potential mediator of antidepressant response and a target through which fluoxetine may exert metabolic effects. Further translational and prospective clinical studies are needed to determine whether these microbiota-related effects are directly induced by fluoxetine and whether they contribute to SSRI-associated changes in body weight.

Importantly, changes in microbial composition and microbial functional activity should not be considered interchangeable. Partial normalization of microbial composition observed following escitalopram or fluoxetine treatment does not necessarily imply complete restoration of microbial metabolic function. This distinction is particularly relevant when considering the potential role of the gut microbiota in SSRI-associated metabolic effects. The available clinical and preclinical evidence regarding fluoxetine-associated alterations in the gut microbiota and their potential relationship with antidepressant treatment response is summarized in Table 3.

Taken together, the available evidence indicates that sertraline, escitalopram, and fluoxetine can modulate both the composition and functional activity of the gut microbiota, although the nature and strength of these effects differ substantially between individual SSRIs. Sertraline is characterized predominantly by direct antimicrobial and microbiota-modulating effects demonstrated in vitro and in animal models, whereas evidence for escitalopram more consistently points toward associations between microbiota composition, microbial metabolic pathways, and antidepressant treatment response. Fluoxetine, in turn, has been associated with broader changes in microbial diversity and metabolic activity, with preclinical studies providing preliminary support for a mediating role of the gut microbiota in its antidepressant effects. Importantly, evidence directly linking these microbiota alterations to SSRI-associated weight gain remains limited, with most available findings being preclinical or indirect. A comparative overview of the main microbiota-related effects of the three SSRIs and their potential relevance to antidepressant efficacy and body weight is presented in Table 4.

## 4. Gut Microbiota as a Regulator of Body Weight Homeostasis

Alterations in the composition and function of the gut microbiota have been shown to influence gut–brain axis signaling, promote inflammatory processes, and disrupt neurotransmitter metabolism, thereby contributing to the development of depressive symptoms [46]. Patients with depression exhibit a reduced abundance of bacteria capable of producing neuroactive metabolites, including SCFAs, together with alterations in the microbial capacity to synthesize and metabolize compounds involved in nervous system function, particularly γ-aminobutyric acid (GABA) and tryptophan-derived metabolites [46]. Gut dysbiosis also compromises intestinal barrier integrity, resulting in increased intestinal permeability and facilitating the translocation of LPS and other bacterial components into the systemic circulation, where they activate the host immune response [47]. This process leads to metabolic endotoxemia, triggering LPS-induced chronic low-grade inflammation characterized by elevated circulating concentrations of pro-inflammatory cytokines, including interleukin-6 (IL-6), interleukin-1β (IL-1β), and tumor necrosis factor-α (TNF-α). These inflammatory mediators can cross the blood–brain barrier and directly affect the CNS, impairing neuroplasticity and disrupting neurotransmitter metabolism [47,48]. Furthermore, activation of inflammatory pathways enhances tryptophan metabolism through the kynurenine pathway at the expense of serotonin synthesis, resulting in increased production of neuroactive kynurenine metabolites, some of which exert neurotoxic effects and have been associated with greater severity of depressive symptoms [48,49]. Simultaneously, depletion of SCFA-producing bacteria, particularly butyrate-producing species, may impair intestinal barrier function, immune regulation, and brain-derived neurotrophic factor (BDNF) expression [47,48,49]. The gut microbiota is a complex ecosystem composed of bacteria, archaea, viruses, and fungi inhabiting the human gastrointestinal tract. It is estimated that the human intestine harbors approximately 10^13^–10^14^ colony-forming units (CFU) of microorganisms [50]. The composition and abundance of these microbial communities vary according to physiological status, anatomical location within the gastrointestinal tract, and host health. In the upper small intestine, bacterial density ranges from approximately 10^3^ to 10^4^ CFU/g of intestinal content and is dominated by members of the genera *Bacteroides*, *Streptococcus*, *Lactobacillus*, *Enterococcus*, *Clostridium*, and *Veillonella*, as well as bacteria belonging to the family *Enterobacteriaceae*. Bacterial density gradually increases along the gastrointestinal tract, reaching approximately 10^7^–10^8^ CFU/g in the ileum and 10^10^–10^12^ CFU/g of feces in the colon. Owing to the relatively slow intestinal transit, the colon harbors the most diverse microbial community, including members of the genera *Bacteroides*, *Clostridium*, *Bifidobacterium*, *Enterococcus*, *Eubacterium*, *Fusobacterium*, *Peptostreptococcus*, *Ruminococcus*, *Streptococcus*, and *Bacillus* [51]. However, defining a universal “healthy” gut microbiota remains challenging because microbial composition is influenced by numerous factors, including intestinal location, luminal pH, oxygen availability, intestinal transit time, and host-derived secretions [51]. Evidence indicates that the gut microbiota functions not only as a protective barrier against pathogens and a facilitator of nutrient digestion but also as an active metabolic organ involved in regulating energy homeostasis, glucose and lipid metabolism, immune function, and bidirectional communication between the gastrointestinal tract and the CNS through the gut–brain axis [52]. Alterations in microbial composition and function (gut dysbiosis) have been implicated in the pathogenesis of obesity, insulin resistance, and other metabolic disorders by influencing energy harvest, appetite regulation, and adipose tissue accumulation [53]. The effects of the gut microbiota on body weight regulation are multifactorial and involve modulation of dietary energy extraction, intestinal barrier integrity, chronic low-grade inflammation, bile acid metabolism, and the production of microbial metabolites that interact with host cells [52,53]. Among these metabolites, SCFAs are considered key mediators of host–microbiota communication. Produced through bacterial fermentation of dietary fiber in the colon, SCFAs represent one of the principal mechanisms through which the gut microbiota influences host physiology [54]. Acetate, propionate, and butyrate account for more than 95% of total SCFAs present in the intestinal lumen. Their concentrations depend primarily on dietary fiber intake, the type of fermentable polysaccharides consumed, and the composition of the gut microbiota. SCFAs function as signaling molecules by activating G protein-coupled receptors (GPCRs), particularly free fatty acid receptor 2 (FFAR2/GPR43), FFAR3 (GPR41), and GPR109A, thereby regulating the secretion of gut hormones involved in appetite and energy homeostasis, including glucagon-like peptide-1 (GLP-1) and peptide YY (PYY). In addition to their metabolic functions, SCFAs exert potent immunomodulatory effects. They regulate immune responses through both GPCR activation and inhibition of histone deacetylases (HDACs), thereby influencing the expression of genes involved in inflammatory pathways. Consequently, SCFAs promote the differentiation of regulatory T cells (Tregs), suppress the production of pro-inflammatory cytokines, and support immune tolerance within the intestinal mucosa. These mechanisms contribute not only to maintaining gastrointestinal homeostasis but also to regulating systemic immune responses [54,55]. SCFAs should be regarded not only as metabolic products of the gut microbiota but also as important modulators of microbial community structure and function. Diets rich in fermentable dietary fiber promote the growth of SCFA-producing bacteria, particularly *Faecalibacterium*, *Roseburia*, and *Bifidobacterium*, while simultaneously facilitating microbial cross-feeding, a process in which metabolites produced by one bacterial species serve as substrates for another. The resulting SCFAs shape the intestinal environment by lowering luminal pH, inhibiting the growth of potentially pathogenic microorganisms, and contributing to the stability and resilience of the gut microbial ecosystem [54,55].

### 4.1. Short-Chain Fatty Acids in Body Weight Regulation

SCFAs, particularly acetate, propionate, and butyrate, are major metabolites generated by the bacterial fermentation of dietary fiber and other non-digestible carbohydrates in the colon. Together, these three SCFAs account for more than 95% of the SCFA pool in the intestinal lumen, although their production and biological effects depend on dietary composition, substrate availability, gut microbial composition, and host metabolic characteristics [52,53,54,55]. Beyond serving as metabolic substrates, SCFAs function as signaling molecules that mediate communication between the gut microbiota and the host. Their effects are mediated predominantly through G protein-coupled receptors, including FFAR2/GPR43, FFAR3/GPR41, and GPR109A, as well as through epigenetic mechanisms such as histone deacetylase inhibition. Through these pathways, SCFAs can influence intestinal barrier integrity, immune responses, adipose tissue metabolism, and enteroendocrine signaling, including the secretion of GLP-1 and PYY, which are important regulators of appetite and energy homeostasis [52,53,54,55,56,57,58].

Among individual SCFAs, butyrate has been most consistently associated with beneficial effects on intestinal and metabolic homeostasis. It is utilized predominantly by colonocytes and contributes to the maintenance of epithelial integrity by supporting tight-junction expression and mucus production. Butyrate also exerts anti-inflammatory and metabolic effects through GPCR-dependent signaling and histone deacetylase inhibition, including modulation of mitochondrial function, fatty acid oxidation, insulin sensitivity, and adipose tissue metabolism [56,57,58]. Experimental studies have generally demonstrated reductions in body weight gain and adipose tissue accumulation following butyrate supplementation, whereas findings from human studies have been less consistent. Available clinical evidence suggests that metabolic effects may be modest and highly dependent on baseline microbiota composition, dietary fiber intake, metabolic status, and individual butyrate exposure [56,57,58,59,60]. Thus, although butyrate represents a biologically plausible mediator of microbiota–host metabolic communication, its independent contribution to long-term body weight regulation in humans remains incompletely defined.

Propionate may be particularly relevant to appetite regulation because of its effects on enteroendocrine signaling. Following colonic absorption, propionate is transported predominantly to the liver, while a smaller proportion reaches the systemic circulation. Through FFAR2 and FFAR3 activation, propionate can stimulate GLP-1 and PYY secretion and thereby influence postprandial satiety and energy intake [55,60,61,62,63]. Experimental and interventional studies have provided evidence that increased colonic delivery of propionate enhances GLP-1 and PYY secretion and may reduce subsequent energy intake [62]. However, the evidence for sustained effects on body weight is less consistent. Although initial randomized trials using an inulin–propionate ester reported favorable effects on appetite and long-term weight gain, the subsequent multicenter iPREVENT trial did not demonstrate superiority over inulin alone for preventing weight gain or improving most metabolic outcomes [62,64,65]. These findings suggest that the metabolic effects of propionate are context-dependent and may vary according to the underlying microbiota, dietary environment, intervention characteristics, and host metabolic phenotype [56,57,58].

Acetate is the most abundant SCFA and is produced predominantly through the fermentation of dietary fiber and resistant starch. Compared with butyrate and propionate, a larger proportion of acetate reaches the systemic circulation, allowing it to interact with peripheral tissues and the central nervous system [55,63]. Acetate activates FFAR2 and FFAR3 and may influence enteroendocrine hormone secretion, adipose tissue metabolism, lipid oxidation, and inflammatory responses. Experimental studies have also suggested that acetate may participate in central regulation of appetite. However, the reported metabolic effects are inconsistent and appear to depend substantially on the source and concentration of acetate, route and duration of administration, and experimental model [55,63,66,67]. Consequently, current evidence does not support a clearly defined independent role for acetate in long-term body weight reduction in humans. Rather, its effects are likely to reflect interactions with other SCFAs, dietary substrates, the gut microbiota, and host metabolic pathways [55,63].

Importantly, the established metabolic effects of individual SCFAs should be distinguished from the evidence linking specific SCFAs to SSRI-associated weight gain. Although alterations in gut microbial composition and metabolic activity during SSRI treatment could theoretically influence SCFA production, direct evidence demonstrating that individual SSRIs consistently alter specific SCFAs in humans remains limited. Similarly, no prospective clinical studies have simultaneously evaluated SSRI exposure, changes in individual SCFA concentrations or production, enteroendocrine signaling, and subsequent changes in body weight. Therefore, evidence from studies investigating the metabolic effects of butyrate, propionate, or acetate in other populations cannot be interpreted as direct evidence of an SSRI–SCFA–weight gain pathway.

From the perspective of SSRI-associated weight gain, the relevance of SCFAs therefore lies primarily in their potential role as functional mediators of microbiota–host metabolic communication, rather than in the independent effects of any single SCFA. A reduction in microbial capacity for SCFA production could theoretically impair FFAR2/FFAR3-mediated signaling, alter GLP-1 and PYY secretion, compromise intestinal barrier function, and modify inflammatory and metabolic pathways involved in energy homeostasis [54,55,62]. However, this proposed sequence remains mechanistic and hypothetical. The current evidence supports the biological plausibility of an SCFA-mediated pathway but does not establish that SSRI-induced alterations in SCFA production are responsible for weight gain during antidepressant therapy. Accordingly, individual SCFAs should be considered within the broader context of microbiota functional activity, dietary substrate availability, intestinal barrier integrity, and host metabolic responses rather than as isolated determinants of SSRI-associated changes in body weight.

### 4.2. Gut Microbiota and Energy Homeostasis

One of the mechanisms through which the gut microbiota may influence body weight regulation is the modulation of energy harvest from the diet. Intestinal microorganisms possess a broad repertoire of enzymes, including glycosidases, lyases, and hydrolases, that are not encoded by the human genome. These enzymes enable the degradation of non-digestible polysaccharides, resistant starch, and other complex carbohydrates that escape digestion in the small intestine. The resulting fermentation products are largely converted into SCFAs, which, following absorption by the intestinal epithelium, serve not only as an additional energy source for the host but also as important signaling molecules regulating multiple metabolic processes [68,69]. The energy acquisition hypothesis was proposed based on the observation that the gut microbiota of obese mice is characterized by an increased metabolic capacity for polysaccharide degradation and increased production of fermentation-derived metabolites. Transfer of this microbiota into germ-free recipient mice resulted in greater adipose tissue accumulation despite comparable energy intake, suggesting that the gut microbiota can modulate host energy utilization and storage [70]. However, more recent metagenomic and metabolomic studies indicate that the contribution of the gut microbiota to energy balance cannot be explained solely by enhanced caloric extraction from otherwise indigestible dietary substrates. SCFAs provide only a modest proportion of total daily energy intake—estimated at approximately 5–10% in individuals consuming a typical Western diet—and their energetic value is approximately 1.5 kcal/g. These findings suggest that the effects of SCFAs on body weight regulation are driven predominantly by their signaling properties rather than by their direct contribution to energy supply. Current evidence indicates that the metabolic effects of SCFAs arise from their involvement in an intricate network of regulatory pathways, including stimulation of GLP-1 and PYY secretion, modulation of bile acid metabolism, maintenance of intestinal barrier integrity, regulation of chronic low-grade inflammation, adipose tissue function, and energy expenditure. Consequently, enhanced energy harvest should be regarded as only one component of the broader metabolic interactions between the gut microbiota and the host, rather than as a single or dominant mechanism underlying obesity development [69]. Accumulating evidence indicates that the influence of the gut microbiota on body weight regulation depends primarily on its metabolic activity and its ability to modulate host immune responses. Functional alterations within the microbiome lead to changes in the production of microbial metabolites, impaired intestinal barrier integrity, and persistent activation of inflammatory pathways, thereby promoting insulin resistance and disturbances in energy homeostasis. Increasingly, studies suggest that these effects are driven less by changes in microbial taxonomic composition than by alterations in the functional capacity of the microbiome and its interactions with the host [54,55,68,69]. Although modulation of the gut microbiota represents a promising target for dietary and pharmacological interventions aimed at preventing or treating obesity, the underlying mechanisms remain incompletely understood and require further investigation.

### 4.3. Metabolic Endotoxemia and Chronic Low-Grade Inflammation

Chronic low-grade inflammation (metaflammation) represents one of the key pathophysiological mechanisms linking gut microbiota dysfunction with the development of obesity, insulin resistance, and other cardiometabolic disorders through metabolic endotoxemia. Current evidence indicates that the metabolic activity of the gut microbiota, rather than taxonomic composition alone, plays a central role in this process. Alterations in microbial metabolic function compromise intestinal barrier integrity, reduce the production of immunoregulatory metabolites, and promote persistent activation of the innate immune system [52,54,55]. One of the earliest consequences of gut dysbiosis is increased intestinal permeability. A reduction in SCFA-producing bacteria, particularly butyrate-producing species, decreases the expression of tight junction proteins, including claudins, occludin, and zonula occludens-1 (ZO-1), weakens the mucus layer, and impairs colonocyte function. Consequently, LPS and other microbe-associated molecular patterns (MAMPs) translocate into the portal and systemic circulation. This phenomenon, known as metabolic endotoxemia, is recognized as one of the principal triggers of chronic activation of the innate immune system in obesity [52,54,55]. Circulating LPS activates the TLR4–CD14–MD2 receptor complex expressed on macrophages, monocytes, dendritic cells, adipocytes, and hepatocytes. Activation of this signaling pathway induces the transcription factors nuclear factor kappa B (NF-κB) and activator protein-1 (AP-1), c-Jun N-terminal kinase (JNK), and the NLRP3 inflammasome, resulting in increased production of pro-inflammatory cytokines, particularly TNF-α, IL-1β, and IL-6. Sustained activation of these inflammatory pathways promotes monocyte recruitment into adipose tissue and drives macrophage polarization toward the pro-inflammatory M1 phenotype, thereby perpetuating local inflammation and progressive adipocyte metabolic dysfunction [54,71,72]. Adipose tissue functions as an active immunometabolic organ in which adipocytes closely interact with immune cells. In obesity, increased infiltration of macrophages, T lymphocytes, and other immune cells establishes a persistent pro-inflammatory microenvironment. This is accompanied by disruption of the balance between pro-inflammatory and pro-resolving mechanisms, leading to chronic inflammation and progressive metabolic impairment. Emerging evidence suggests that defective endogenous mechanisms responsible for the resolution of inflammation, including specialized pro-resolving mediators (SPMs), contribute to obesity pathogenesis to a similar extent as excessive inflammatory activation itself [72]. Persistent immune activation directly impairs insulin signaling. Pro-inflammatory cytokines and TLR4-dependent kinases induce serine phosphorylation of insulin receptor substrate-1 (IRS-1), thereby inhibiting activation of the phosphoinositide 3-kinase/protein kinase B (PI3K/Akt) signaling pathway and reducing GLUT4 translocation to the plasma membrane of skeletal muscle cells and adipocytes. As a result, glucose uptake by peripheral tissues is diminished, leading to insulin resistance and secondary disturbances in lipid metabolism. Simultaneously, the adipokine profile becomes dysregulated, with increased secretion of leptin and resistin and reduced adiponectin production, further amplifying inflammation and metabolic dysfunction [55,71,72]. The relationship between gut dysbiosis, metabolic endotoxemia, and obesity is bidirectional. High-fat diets and excess adiposity impair intestinal barrier function and alter the metabolic activity of the gut microbiota, whereas sustained immune activation exacerbates insulin resistance and adipose tissue dysfunction, creating a self-perpetuating vicious cycle. Increasing evidence indicates that immunometabolic homeostasis is influenced not only by exposure to LPS but also by alterations in the production of SCFAs, secondary bile acids, and tryptophan-derived metabolites, which collectively regulate host immune and metabolic responses [52,54,55,71,72].

## 5. Gut Microbiota as a Potential Mediator of SSRI-Associated Weight Gain

Weight gain is one of the most frequently reported adverse effects of long-term SSRI therapy; however, the mechanisms underlying its development remain incompletely understood. Current pathophysiological concepts primarily focus on adaptive changes in serotonergic neurotransmission, including alterations in appetite regulation, energy expenditure, and hypothalamic pathways involved in energy homeostasis. Nevertheless, these mechanisms do not adequately explain the marked interindividual variability in metabolic responses observed among patients receiving the same class of antidepressants or the delayed onset of weight gain, which often develops only after several months of treatment despite the pharmacodynamic effects of SSRIs being established much earlier [7,9,73]. In recent years, growing evidence has identified the gut microbiota as an important determinant of host responses to pharmacotherapy. Numerous studies have demonstrated that a wide range of medications, including antidepressants, can alter both the composition and metabolic activity of the gut microbiome, while intestinal microorganisms, in turn, influence drug metabolism, bioavailability, and clinical response. The emerging field of pharmacomicrobiomics suggests that these bidirectional interactions may also contribute to drug-induced metabolic adverse effects; however, the underlying biological mechanisms remain largely unresolved [23,68,69]. Based on the available evidence, we propose that the gut microbiota may represent a previously underappreciated mediator of SSRI-associated weight gain. Specifically, SSRI-induced alterations in microbial metabolic activity may influence appetite regulation, intestinal barrier integrity, and host immunometabolic responses, ultimately promoting a positive energy balance and the gradual development of metabolic disturbances that favor weight gain. Importantly, this proposed mechanism should be regarded as a conceptual framework integrating evidence from several independent fields of research rather than as a confirmed pathophysiological pathway. To date, no clinical studies have comprehensively evaluated all components of this hypothesis within a single experimental setting.

### 5.1. Disruption of the Metabolic Potential of the Gut Microbiota and the SCFA–GLP-1/PYY Axis as a Hypothetical Mechanism of SSRI-Associated Weight Gain

One of the most biologically plausible mechanisms that may explain the association between long-term SSRI therapy and weight gain is the disruption of the metabolic potential of the gut microbiota. Unlike the traditional perspective, which focuses primarily on alterations in microbial taxonomic composition, emerging evidence suggests that the functional activity of the gut microbiome plays a more critical role in maintaining host metabolic homeostasis. This functional capacity includes the fermentation of dietary fiber, the production of SCFAs, and the regulation of signaling pathways between the intestine and peripheral organs [7,9,74]. Experimental evidence indicates that certain SSRIs may influence the gut microbiota both directly, through the antimicrobial properties of specific compounds, and indirectly by altering gastrointestinal motility, intestinal serotonin availability, and the metabolic environment of the gut. These alterations may not only modify microbial composition but also reduce the fermentative capacity of the gut microbiota, leading to decreased production of regulatory bacterial metabolites, particularly butyrate and propionate [17,18,19,72]. Reduced SCFA availability may hypothetically impair the activation of FFAR2 and FFAR3 expressed on enteroendocrine L cells, resulting in diminished secretion of GLP-1 and PYY. These hormones play a central role in the short-term regulation of energy balance by promoting satiety, delaying gastric emptying, and modulating postprandial insulin secretion. Consequently, disruption of the SCFA–GLP-1/PYY signaling axis may contribute to increased energy intake and the gradual development of a positive energy balance [9,62,74]. Notably, interventional studies using IPE have demonstrated that targeted delivery of propionate to the colon increases postprandial GLP-1 and PYY secretion, reduces energy intake, and attenuates weight gain in individuals with overweight. Although these findings were not obtained in patients receiving SSRI therapy, they support the biological plausibility of a mechanistic link between microbial metabolic activity and appetite regulation [57,62]. To date, no prospective clinical studies have simultaneously evaluated the effects of SSRI therapy on gut microbiota function, SCFA production, enteroendocrine hormone secretion, and changes in body weight. Therefore, the mechanism proposed here remains hypothetical and represents an attempt to integrate evidence derived from several independent fields of research. At present, there is insufficient evidence to conclude that altered metabolic activity of the gut microbiota directly contributes to SSRI-associated metabolic adverse effects. Nevertheless, the available evidence suggests that this hypothesis is biologically coherent and warrants further investigation in well-designed translational studies.

### 5.2. Intestinal Barrier Dysfunction and Activation of Immunometabolic Pathways as a Potential Mechanism of SSRI-Associated Weight Gain

Another, potentially complementary, mechanism linking SSRI therapy with the development of metabolic disturbances may involve gut microbiota-driven disruption of intestinal barrier integrity and the subsequent activation of immunometabolic pathways. This hypothesis is supported by numerous observations demonstrating that the integrity of the intestinal epithelium is a key determinant of host metabolic homeostasis, whereas its impairment promotes the development of chronic low-grade inflammation, a hallmark of obesity and metabolic syndrome [54,55,68,69]. Under physiological conditions, the gut microbiota contributes to the maintenance of intestinal barrier function by regulating the expression of tight junction proteins, stimulating mucus production, and generating microbial metabolites, particularly butyrate, which serves as the primary energy source for colonocytes. Impairment of the metabolic activity of the gut microbiota may weaken these protective mechanisms, resulting in increased intestinal permeability and facilitating the translocation of LPS and other pro-inflammatory bacterial components into the portal and systemic circulation [54,57]. Chronic exposure of the immune system to low concentrations of LPS, referred to as metabolic endotoxemia, activates Toll-like receptors, particularly TLR4, leading to the activation of NF-κB-dependent intracellular signaling pathways. Consequently, the production of pro-inflammatory cytokines, including TNF-α, IL-1β, and IL-6, is increased. These cytokines impair insulin signaling in adipose tissue, skeletal muscle, and the liver. Persistent chronic low-grade inflammation is now recognized as one of the principal pathophysiological mechanisms underlying insulin resistance and adipose tissue dysfunction [55,68,72]. In the context of SSRI therapy, it can be hypothesized that the alterations in gut microbiota composition and metabolic activity reported in some studies may indirectly impair intestinal barrier function and promote metabolic endotoxemia. Although this mechanism is biologically plausible, direct confirmation requires clinical studies simultaneously assessing changes in the gut microbiota, markers of intestinal permeability, circulating LPS concentrations, inflammatory pathway activation, and metabolic outcomes in patients receiving SSRI treatment. At present, the available evidence does not allow definitive conclusions regarding the role of this pathway as a mediator of SSRI-associated weight gain. Nevertheless, its consistency with the current immunometabolic model of obesity provides a strong rationale for further experimental and translational investigation.

### 5.3. Modulation of Tryptophan Metabolism and the Gut–Brain Axis as a Potential Mechanism Underlying Interindividual Variability in the Metabolic Response to SSRI Therapy

In addition to impaired SCFA-dependent signaling and activation of immunometabolic pathways, modulation of tryptophan metabolism by the gut microbiota may represent another mechanism linking SSRI therapy with body weight regulation. This hypothesis is based on the observation that intestinal microorganisms play a pivotal role in regulating tryptophan availability and directing its metabolic fate, thereby influencing the production of a wide range of biologically active compounds, including indoles, kynurenine pathway metabolites, and gut-derived serotonin [55,72,75]. An increasing body of evidence indicates that the functional activity of the gut microbiota contributes to maintaining the balance between the major pathways of tryptophan metabolism. The resulting microbial metabolites interact with multiple host targets, including the aryl hydrocarbon receptor (AhR), immune cells, neurons of the enteric nervous system, and enteroendocrine cells, thereby participating in the regulation of intestinal barrier integrity, inflammatory responses, and gut–brain axis communication. Dysregulation of these processes has been implicated in both metabolic and neuropsychiatric disorders, suggesting the existence of shared immunometabolic mechanisms underlying these seemingly distinct conditions [47,55,72]. It can therefore be hypothesized that SSRI-induced alterations in the metabolic activity of the gut microbiota may modify the profile of tryptophan-derived metabolites, thereby influencing signaling within the gut–brain axis. Such changes could, in turn, affect appetite regulation, food preferences, and the neuroendocrine pathways responsible for maintaining energy homeostasis. This mechanism is particularly intriguing in the context of the considerable interindividual variability observed in the metabolic response to antidepressant therapy, as differences in gut microbiota composition and function may influence both the availability of tryptophan metabolites and host sensitivity to their biological effects [69,72,75]. However, most of the available evidence originates from preclinical studies or from investigations examining depression, obesity, and gut microbiota independently. Consequently, there are currently no clinical studies directly evaluating the effects of SSRI therapy on microbial tryptophan metabolism and its relationship with changes in body weight. Therefore, this hypothesis remains speculative and requires validation in prospective translational studies integrating comprehensive analyses of the gut microbiome, metabolome, and metabolic phenotype.

### 5.4. Proposed Mechanistic Model of the Role of the Gut Microbiota in SSRI-Associated Weight Gain

**Figure 1:**
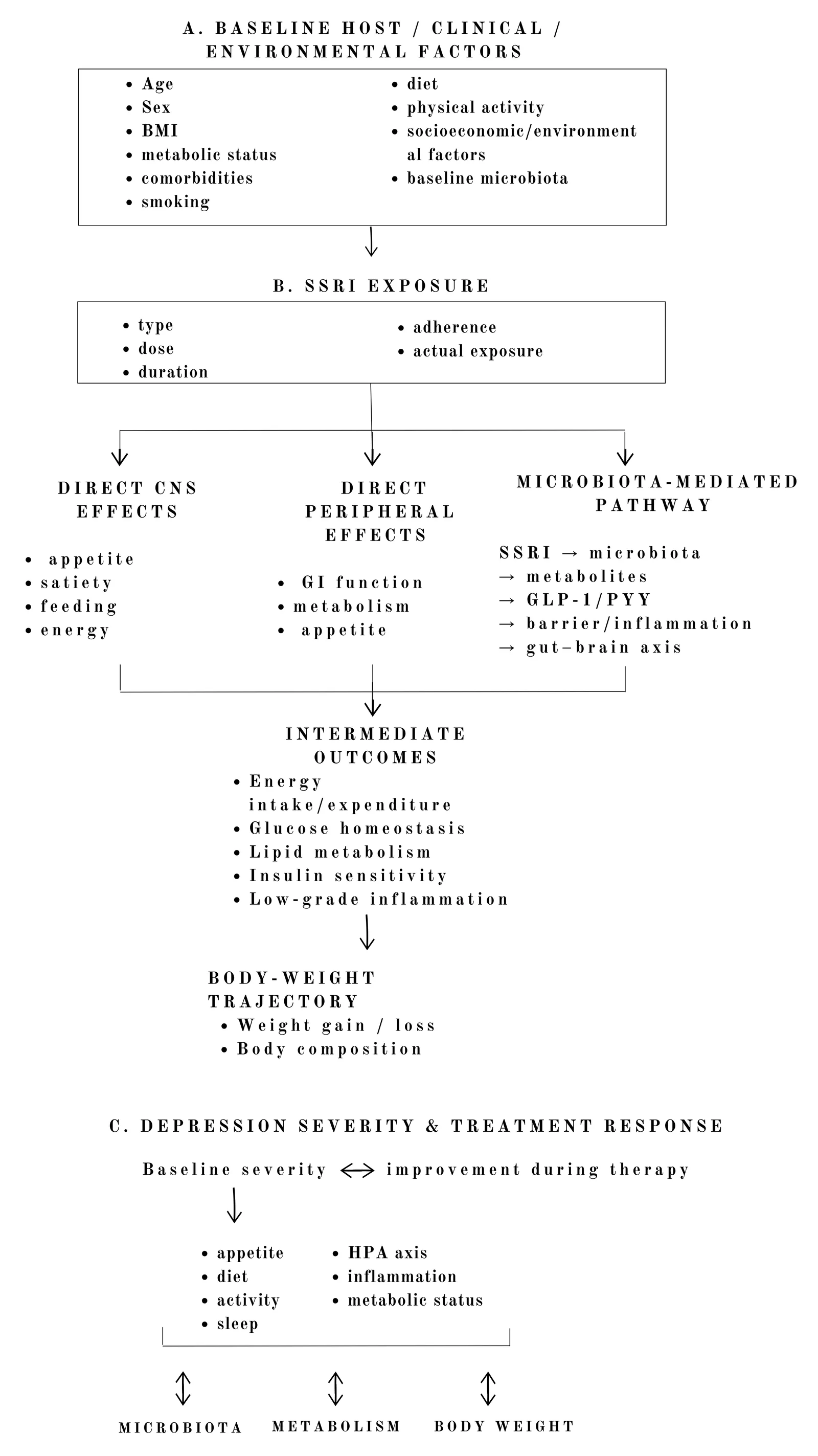
Proposed mechanistic model of the role of the gut microbiota in SSRI-associated weight gain. BMI, body mass index; CNS, central nervous system; GI, gastrointestinal; GLP-1, glucagon-like peptide-1; HPA, hypothalamic–pituitary–adrenal; PYY, peptide YY; SSRI, selective serotonin reuptake inhibitor.

The mechanisms discussed above should not be considered mutually exclusive but rather as interconnected components of a complex biological network that may collectively regulate the host metabolic response to long-term SSRI therapy. Current concepts of gut microbiota function indicate that its influence on host physiology is multidimensional, encompassing the simultaneous modulation of metabolic, immunological, and neuroendocrine processes. Consequently, it is unlikely that a single mechanism is solely responsible for the development of weight gain observed in a subset of patients receiving SSRI treatment [54,55,68,69,72]. In the proposed model, the initiating event is an SSRI-induced alteration in the functional activity of the gut microbiota. Impairment of the metabolic potential of the gut microbiome may simultaneously reduce the production of regulatory bacterial metabolites, compromise intestinal barrier integrity, and alter tryptophan metabolism. These processes are closely interconnected and may reinforce one another, ultimately disrupting host immunometabolic homeostasis [47,54,55,68,69,72]. Reduced production of SCFAs may attenuate the secretion of enteroendocrine hormones involved in satiety regulation, whereas increased intestinal permeability may promote metabolic endotoxemia and the persistent activation of inflammatory pathways. Concurrently, alterations in microbial tryptophan metabolism may influence gut–brain axis signaling, thereby modulating appetite regulation and feeding behavior. Collectively, these mechanisms may contribute to the development of a sustained positive energy balance, progressive insulin resistance, and ultimately body weight gain [47,54,55,69,72,75]. An important assumption of the proposed model is that the gut microbiota is not the sole determinant of the metabolic adverse effects associated with SSRI therapy but rather a potential mediator capable of modifying the host response to treatment. Accordingly, the impact of SSRI therapy on body weight is likely to depend on multiple interacting factors, including the baseline composition and functional capacity of the gut microbiota, dietary patterns, dietary fiber intake, physical activity, genetic predisposition, and other environmental influences. Such a multifactorial model may partly explain the substantial interindividual variability in weight gain observed among patients receiving the same class of antidepressants [54,55,68,69,72]. It should be emphasized that the proposed model is conceptual and represents an attempt to integrate evidence derived from microbiology, immunology, endocrinology, and psychopharmacology. At present, prospective studies simultaneously evaluating gut microbiota composition and function, microbial metabolite profiles, gut–brain axis activity, and metabolic outcomes in patients undergoing SSRI therapy are lacking. Validation of this hypothesis will therefore require well-designed multicenter translational studies integrating comprehensive analyses of the microbiome, metabolome, and clinical phenotype. The proposed mechanistic model is presented in Figure 1. The model is conceptual and hypothesis-generating rather than a validated causal framework. The microbiota-mediated pathway linking SSRI exposure with body-weight changes has not been directly demonstrated in longitudinal human studies.

### 5.5. Clinical Implications, Strengths and Limitations of the Proposed Model, and Future Research Directions

The proposed mechanistic model provides a conceptual framework integrating evidence from microbiology, immunology, endocrinology, metabolism, and psychopharmacology to describe potential pathways linking SSRI therapy, gut microbiota alterations, metabolic regulation, and body weight. A major strength of this approach is the integration of several complementary mechanisms, including changes in microbial metabolite production, intestinal barrier integrity, tryptophan metabolism, gut–brain axis signaling, appetite regulation, inflammation, and insulin sensitivity. Importantly, the model emphasizes that SSRI-associated weight gain is likely to result from interactions among multiple biological, metabolic, and environmental factors rather than from a single mechanism [54,55,68,69,72]. Within this framework, assessment of gut microbiota composition and functional capacity may, in the future, contribute to the identification of individuals at increased risk of metabolic adverse effects during antidepressant treatment and potentially support more individualized therapeutic strategies [54,55,68,69].

At the same time, several limitations should be considered when interpreting the proposed framework. First, this study is a narrative review rather than a systematic review or meta-analysis. Although the literature search included PubMed/MEDLINE, Scopus, and Web of Science, and priority was given to systematic reviews, meta-analyses, randomized controlled trials, prospective clinical studies, and well-designed experimental investigations, the available evidence remains heterogeneous with respect to study design, populations, interventions, and assessed outcomes. Therefore, the proposed model should be considered hypothesis-generating rather than a validated causal framework. The individual components of the proposed pathways are supported to varying degrees, but their integration into a single causal sequence has not yet been directly demonstrated in humans.

A second important limitation is the limited availability of longitudinal human evidence. Although clinical and experimental studies indicate that SSRIs may influence the composition and functional activity of the gut microbiota, it remains unclear whether these changes represent a direct pharmacological effect of SSRI treatment or occur secondarily to clinical improvement and associated changes in appetite, dietary composition, physical activity, sleep, inflammatory status, or metabolic parameters [36]. Furthermore, available clinical studies have not directly demonstrated that microbiota alterations mediate SSRI-associated weight gain. Consequently, the temporal and causal relationships between SSRI exposure, microbiota changes, microbial metabolites, metabolic alterations, and subsequent weight gain remain uncertain [36,37,38,39].

Another important limitation concerns potential moderators and confounding factors that cannot be fully addressed by the current model. The impact of SSRI therapy on body weight is likely to depend on baseline gut microbiota composition and functional capacity, dietary patterns, dietary fiber intake, physical activity, genetic predisposition, and other environmental influences [54,55,68,69,72]. In addition, baseline metabolic status, comorbidities, and concomitant medications may independently influence both the gut microbiota and metabolic outcomes [54,55,68,69]. These factors may therefore contribute to the substantial interindividual variability observed in weight gain during SSRI treatment and complicate the interpretation of associations between antidepressant exposure, microbiota alterations, and metabolic outcomes. The current evidence does not allow these factors to be fully separated from potential direct effects of SSRIs.

A further limitation is the predominance of mechanistic and preclinical evidence for several of the proposed pathways. For example, evidence concerning the metabolic effects of SCFAs and their potential involvement in body weight regulation has been derived predominantly from experimental studies, while available human findings remain less consistent and indicate substantial interindividual variability [56,57,58]. Similarly, although changes in microbial tryptophan metabolism and gut–brain axis signaling provide a biologically plausible mechanism, their specific contribution to SSRI-associated weight gain has not been established in prospective human studies [36,37,38,39]. Thus, biological plausibility should not be interpreted as evidence of clinical causality.

Despite these limitations, the proposed framework may help identify specific questions for future translational research. Future studies should move beyond evaluating individual components of the proposed pathway and prospectively investigate the SSRI–microbiota–metabolic axis as an integrated system. A particularly informative approach would involve multicentre prospective longitudinal studies enrolling adults with depression who are initiating SSRI treatment. Participants should be assessed before or at the initiation of treatment and followed at predefined time points during therapy. Repeated measurements should include body weight and body composition, metabolic parameters, depressive symptom severity, dietary intake, physical activity, sleep, and concomitant medication use. Serial stool samples should be collected to characterize gut microbiota composition and functional capacity using metagenomic and, where feasible, metatranscriptomic approaches. Metabolomic analyses should additionally assess relevant microbiota-derived metabolites, including SCFAs and tryptophan-derived metabolites [54,55,68,69]. Such an integrated longitudinal design would help determine whether microbiota alterations precede metabolic changes and weight gain, occur concurrently with them, or instead represent secondary consequences of treatment and clinical improvement.

Future studies should also incorporate appropriate comparator groups and systematically account for major potential confounders. Where feasible, comparisons could include patients with depression who do not receive pharmacological treatment and healthy controls, allowing treatment-related changes to be distinguished from alterations associated with depression itself. Analyses should account for baseline body mass index, dietary intake, physical activity, metabolic status, comorbidities, concomitant medications, and baseline microbiota characteristics. Importantly, studies should examine whether specific microbial or metabolomic signatures are associated with subsequent weight gain and whether these profiles modify the metabolic response to different SSRIs. This approach could help determine whether the gut microbiota functions primarily as a mediator, a moderator, or a correlate of SSRI-associated metabolic effects.

Finally, interventional studies will be necessary to establish causality and determine whether microbiota modulation can influence clinically relevant outcomes. Dietary interventions, increased intake of fermentable dietary fiber, and microbiota-targeted approaches involving prebiotics, probiotics, synbiotics, or postbiotics may represent potential adjunctive strategies because they could influence microbial metabolic activity, SCFA production, and intestinal barrier integrity [54,55,57,69]. However, no well-designed clinical trials have yet demonstrated that these interventions reduce the risk of SSRI-associated weight gain. Their clinical use should therefore currently be regarded as promising but experimental and should be evaluated in appropriately designed randomized controlled trials [54,57,69]. Ultimately, combining prospective longitudinal observational studies with mechanistically informed intervention trials may provide the evidence required to determine whether gut microbiota alterations play a causal role in SSRI-associated weight gain and whether this pathway can be targeted to improve the metabolic safety of antidepressant treatment.

### 5.6. Potential Relevance of Dietary and Microbiota-Targeted Interventions to SSRI-Associated Weight Gain

A growing body of evidence indicates that diet is one of the major determinants of gut microbiota composition and metabolic activity, thereby influencing the gut–brain axis, inflammatory pathways, and whole-body energy homeostasis [76]. Dietary patterns modulate the gut microbiota not only by altering its taxonomic composition but also by affecting its metabolic activity, including the production of SCFAs, secondary bile acids, and tryptophan-derived metabolites, all of which play important roles in regulating host metabolism, immune function, and neuroendocrine signaling [76,77]. These findings provide a general biological rationale for investigating whether dietary modulation of the gut microbiota could influence metabolic outcomes during antidepressant treatment. However, evidence derived from studies of diet quality, depression, obesity, or general metabolic health cannot be directly extrapolated to the prevention of SSRI-associated weight gain. To date, no randomized controlled trials have evaluated microbiota-targeted dietary interventions for this specific indication. A recent systematic review by Mötteli et al. found that the available clinical evidence was limited to patients receiving antipsychotic medications, with no eligible studies involving individuals with depression treated with antidepressants [78]. Therefore, the approaches discussed below should be regarded as hypothesis-generating research directions rather than established preventive or therapeutic strategies [76]. A recent systematic review by Mötteli et al. reported that the available clinical evidence is limited to patients receiving antipsychotic medications, with no studies identified in individuals with depression treated with antidepressants. The authors highlighted this as a major knowledge gap and emphasized the need for well-designed clinical trials in this patient population [78].

#### 5.6.1. Dietary Fiber

Dietary fiber is one of the most extensively studied dietary components capable of modulating both the composition and metabolic activity of the gut microbiota. Because it cannot be digested by human enzymes in the small intestine, it undergoes bacterial fermentation in the colon, resulting in the production of SCFAs [79,80]. A systematic review and meta-analysis by Aslam et al., including 23 observational studies involving 181,405 participants as well as randomized intervention trials, demonstrated that higher dietary fiber intake was associated with a significantly lower risk of depression. The authors suggested that these beneficial effects may be mediated through gut microbiota modulation and increased SCFA production; however, the number of intervention studies remains limited, and their findings are inconsistent [81]. The beneficial effects of dietary fiber on the gut microbiota have also been confirmed by meta-analyses of intervention studies conducted in healthy individuals. Fiber supplementation, particularly with fructans and galactooligosaccharides, has been shown to increase the abundance of *Bifidobacterium* and *Lactobacillus*, although it has no significant effect on gut microbial α-diversity. In addition, a systematic review of clinical studies demonstrated that fermentable fiber fractions enhance SCFA production, which may partly explain their beneficial effects on host metabolism and the regulation of inflammatory processes [79,82]. Although dietary fiber can modify gut microbial composition and metabolite production and may support general metabolic health, these findings constitute only indirect evidence in the context of SSRI treatment. No studies have evaluated whether increasing dietary fiber intake prevents or reduces weight gain among patients receiving SSRIs. Its potential role in this setting therefore remains hypothetical and should be assessed in randomized controlled trials specifically designed to evaluate body weight, body composition, appetite, and metabolic outcomes during SSRI therapy [83].

#### 5.6.2. Mediterranean Diet

The Mediterranean diet (MedDiet) is one of the best-characterized dietary patterns shown to exert beneficial effects on metabolic health, inflammatory regulation, and gut–brain axis function. Its biological effects are largely attributed to its high intake of plant-based foods, including vegetables, fruits, legumes, whole grains, nuts, and extra virgin olive oil, which provide dietary fiber, polyphenols, monounsaturated fatty acids, and other bioactive compounds capable of modulating both the composition and metabolic activity of the gut microbiota [84]. In recent years, increasing attention has been paid to the role of the MedDiet in shaping the functional profile of the gut microbiota rather than merely its taxonomic diversity. A systematic review of observational and interventional studies by Khavandegar et al. demonstrated that adherence to the MedDiet may alter gut microbial composition and increase the production of microbiota-derived metabolites, including SCFAs, which contribute to the regulation of energy metabolism, intestinal barrier integrity, and host immune responses [84]. These mechanisms may be particularly relevant in depression, as disturbances of the gut–brain axis are characterized by increased intestinal permeability, chronic immune activation, oxidative stress, and impaired metabolism of neuroactive compounds, including tryptophan metabolites and SCFAs. By increasing the intake of fermentable substrates utilized by commensal bacteria, the MedDiet may promote the growth of microorganisms associated with a favorable metabolic profile, including butyrate-producing bacteria such as Faecalibacterium and Roseburia [84,85]. Clinical evidence also supports a potential role of the MedDiet in alleviating depressive symptoms. Although most studies have not directly assessed changes in the gut microbiota as mediators of therapeutic effects, randomized controlled trials have demonstrated that improving overall diet quality can reduce the severity of depressive symptoms. These benefits are thought to result from multiple mechanisms, including reduced systemic inflammation, improved insulin sensitivity, and potential modulation of the gut–brain axis [86]. In the context of SSRI therapy, the metabolic and microbiota-related effects of the Mediterranean diet provide a plausible rationale for further investigation. However, evidence that this dietary pattern improves depressive symptoms or general metabolic health does not demonstrate that it prevents weight gain caused by SSRIs [87]. No randomized controlled trials have evaluated the Mediterranean diet for the prevention of weight gain in individuals initiating or continuing SSRI therapy. Accordingly, it cannot currently be recommended specifically for this purpose, although it may still be appropriate as part of general evidence-based dietary care.

#### 5.6.3. Potential Role of Prebiotics, Probiotics, Synbiotics, and Postbiotics in Modulating the Gut–Brain Axis During SSRI Therapy

A growing body of evidence suggests that the gut microbiota is an important mediator linking dietary patterns, host metabolism, and CNS function. Consequently, microbiota-targeted interventions, including prebiotics, probiotics, synbiotics, and postbiotics, are being investigated primarily in relation to depressive symptoms and gut–brain axis function. Their potential effects on metabolic outcomes during antidepressant treatment remain largely theoretical [88]. The proposed mechanisms underlying these interventions include modulation of gut microbial composition, increased production of bacterial metabolites—particularly SCFAs—improvement of intestinal barrier integrity, attenuation of immune activation, and regulation of neuroactive compounds, including serotonin, GABA, and metabolites of the kynurenine pathway [89,90]. Among microbiota-targeted interventions, probiotics have been investigated most extensively. Often referred to as psychobiotics, these microorganisms are thought to influence gut–brain axis signaling. Strains belonging primarily to the genera Lactobacillus and Bifidobacterium have demonstrated the potential to modulate immune responses, reduce circulating pro-inflammatory cytokine levels, improve intestinal barrier function, and regulate tryptophan metabolism [89,91]. A meta-analysis of randomized controlled trials conducted by Zhang et al. demonstrated that supplementation with probiotics, prebiotics, or synbiotics was associated with significant improvements in depressive symptoms compared with placebo, with larger reductions in depressive symptom scores observed in some probiotic-containing interventions. These benefits were particularly evident among individuals with mild-to-moderate depression. However, the authors emphasized the considerable heterogeneity in probiotic strains, dosages, and intervention durations, limiting the ability to formulate definitive clinical recommendations [91]. A more recent meta-analysis of randomized controlled trials involving patients with depressive and anxiety disorders also reported significant reductions in depressive symptoms following probiotic supplementation. In contrast, the effects of prebiotics remained inconclusive because of the limited number of available studies and the substantial heterogeneity of the interventions evaluated [92]. To date, clinical research has focused primarily on the effects of probiotics on psychiatric outcomes rather than on the prevention of SSRI-associated weight gain. Any protective effect against SSRI-associated weight gain is currently hypothetical and is inferred from studies examining glucose metabolism, lipid homeostasis, appetite regulation, or inflammation in other populations. Whether these effects occur in patients receiving SSRIs and whether they result in clinically meaningful differences in body weight remain unknown.

Prebiotics are selectively utilized substrates that promote the growth and metabolic activity of beneficial gut microorganisms, thereby enhancing the production of biologically active microbial metabolites. Within the context of the gut–brain axis, particular attention has been given to inulin-type fructans, fructooligosaccharides (FOS), and galactooligosaccharides (GOS), which have been shown to increase the abundance of *Bifidobacterium* species and stimulate SCFA production [89,90].

Synbiotics, defined as combinations of probiotics and prebiotics, may exert synergistic effects by simultaneously delivering beneficial microorganisms and the substrates required to support their metabolic activity. Available evidence suggests that synbiotics may improve depressive symptoms; however, the number of clinical studies remains limited, and further validation is required before they can be recommended as adjunctive therapy for depression [91,92].

Postbiotics, which include inactivated microorganisms, their structural components, and microbiota-derived metabolites, represent an emerging area of microbiome research. Compared with probiotics, postbiotics may offer several advantages, including greater biological stability and the absence of a requirement for gastrointestinal colonization by viable microorganisms [89]. Among postbiotic metabolites, butyrate has attracted particular attention because of its anti-inflammatory properties, its ability to strengthen intestinal barrier function, and its potential role in promoting neuroplasticity through the regulation of histone acetylation-dependent gene expression [90]. Despite promising experimental findings, clinical evidence supporting the use of postbiotics for depression or the prevention of SSRI-related metabolic adverse effects remains insufficient.

Fecal microbiota transplantation (FMT) represents the most intensive approach to gut microbiota modulation and is currently an established treatment for recurrent Clostridioides difficile infection [93]. In recent years, FMT has also attracted interest as a potential therapeutic strategy for metabolic and neuropsychiatric disorders; however, its application in depression remains experimental [94]. Owing to the lack of standardized protocols, challenges in identifying optimal donor microbiota profiles, and the potential risk of unpredictable ecological alterations within the gut microbiome, FMT cannot currently be recommended either as an adjunctive treatment for depression or as a strategy for preventing SSRI-associated weight gain [94]. Overall, dietary fiber, Mediterranean-style dietary patterns, and microbiota-targeted interventions have biological effects that could theoretically influence pathways involved in appetite regulation, inflammation, energy metabolism, and gut–brain axis signaling. However, the available evidence has been derived predominantly from studies of depressive symptoms, general metabolic health, or populations not receiving SSRIs. No randomized controlled trial has demonstrated that any dietary, probiotic, prebiotic, synbiotic, postbiotic, or other microbiota-targeted intervention prevents or reduces SSRI-associated weight gain. These approaches should therefore not be considered established clinical recommendations for this indication. Future randomized controlled trials should evaluate their effects in patients initiating SSRI therapy, while accounting for SSRI type and dose, treatment duration, changes in depression severity, baseline metabolic status, dietary intake, physical activity, and concomitant medications.

## 6. Conclusions

Available experimental and clinical evidence indicates that the gut microbiota is an important regulator of metabolic, immunological, and neuroendocrine processes involved in maintaining energy homeostasis. An increasing body of evidence suggests that long-term SSRI therapy may alter both the composition and functional activity of the gut microbiota. However, the contribution of these alterations to the development of metabolic adverse effects, particularly weight gain, remains poorly understood.

Based on the available evidence, we propose a mechanistic model in which SSRI-induced alterations in gut microbiota function may contribute to body weight regulation through three interconnected biological pathways: (i) reduced production of SCFAs and impaired incretin signaling, (ii) disruption of intestinal barrier integrity leading to metabolic endotoxemia and chronic low-grade inflammation, and (iii) modulation of tryptophan metabolism and gut–brain axis communication. Importantly, this model should be regarded as a conceptual framework integrating current evidence rather than a confirmed pathophysiological mechanism.

Validation of this proposed model will require well-designed prospective translational studies integrating longitudinal analyses of gut microbiota composition and function, microbial metabolite profiles, intestinal barrier integrity, gut–brain axis activity, and metabolic outcomes in patients initiating SSRI therapy. Confirmation of these mechanisms could provide the basis for the development of novel preventive and therapeutic strategies aimed at reducing the metabolic adverse effects of antidepressant treatment through targeted modulation of the gut microbiota within the framework of precision medicine. In parallel, well-designed randomized controlled trials are needed to determine whether microbiota-targeted nutritional interventions can effectively prevent or attenuate SSRI-associated weight gain.

## Figures and Tables

**Table 1:** Summary of evidence regarding the effects of sertraline on the gut microbiota, microbial functions, and their potential relevance to antidepressant efficacy and SSRI-associated weight gain.

Reference	Study Desing	Population/Model	Main Findings	Relevance to Antidepressant Efficacy Evidence	Relevance to SSRI-Associated Weight Gain
McGovern et al. [19]	Review/predominantly experimental evidence	Microbial models	Antimicrobial activity of SSRIs and potential effects on microbial composition and function	Provides biological rationale for microbiota modulation	Indirect; no direct evidence
Liu et al. [22]	In vitro	Bacterial and fungal species	Inhibition of microbial growth following exposure to sertraline	No direct evidence	No direct evidence
McVey Neufeld et al. [24]	In vivo; animal study	Mice	↓ gut microbial α-diversity and selective changes in bacterial taxa; effects persisted after vagotomy	Potentially relevant; antidepressant effects required intact vagal signaling	No direct evidence
McGovern, Caldara, Ahmed, Wang et al. [19,20,25,26]	Predominantly in vitro	Bacteria and fungi	Inhibition of microbial proliferation, adhesion and biofilm formation	Indirect mechanistic relevance	No direct evidence
Ahmed et al. [25]	In vitro	*C. albicans* biofilms	↓ biofilm biomass and metabolic activity; ↓ expression of genes involved in adhesion and maturation	Indirect	No direct evidence
Oliveira et al. [27]	In vitro	Preformed biofilms	Inhibition of established biofilms	Indirect	No direct evidence
Cong, Zhai et al. [28,29]	In vitro	Planktonic fungal cells and biofilms	Synergistic activity of sertraline with fluconazole and amphotericin B	Indirect	No direct evidence
McGovern, Sjöstedt et al. [19,30]	Review/experimental evidence	Microbial models	Effects on membrane homeostasis, metabolism, adaptive capacity and biofilm formation	Mechanistic background	Indirect
Endo, Wang et al. [32,33]	Preclinical/predominantly in vitro	Bacterial models	Changes in efflux pump activity, oxidative stress, membrane integrity and persister-cell formation	Not directly investigated	No direct evidence
Lu et al. [34]	In vitro; bacterial model	Bacterial transformation model	Increased horizontal transfer of antibiotic-resistance genes	Not directly investigated	No direct evidence

↓ decrease.

**Table 2:** Summary of clinical and preclinical evidence on the association between escitalopram, gut microbiota alterations, and antidepressant treatment response.

Reference	Study Desing	Population/Model	Main Findings	Relevance to Antidepressant Efficacy Evidence	Relevance to SSRI-Associated Weight Gain
Shen et al. [36]	Prospective clinical study	Treatment-naive patients with first-episode MDD; healthy controls	Escitalopram partially normalized microbial dysbiosis and diversity; metabolic differences persisted despite clinical improvement.	Associative clinical evidence linking microbiota changes with treatment response.	Not assessed
Wang et al. [37]	Prospective clinical study; multi-omics	110 patients with MDD; escitalopram for 12 weeks	Responders and non-responders differed in microbial and metabolic profiles; tryptophan/indole pathways and IPA were associated with response.	Strongest clinical evidence supporting a potential role of microbiota and microbial metabolites in treatment response.	Not assessed
Jiang et al. [38]	Experimental animal study	Animal model	Escitalopram partially restored eubiosis and altered phospholipid/sphingolipid metabolism.	Preclinical mechanistic support for a role of microbiota function in antidepressant efficacy.	Not assessed.
Duan et al. [39]	Experimental animal study	Animal model	Escitalopram altered microbial composition and metabolic pathways, particularly phospholipid/sphingolipid metabolism.	Preclinical mechanistic support for a role of microbiota function in antidepressant efficacy.	Not assessed

**Table 3:** Summary of clinical and preclinical evidence on the association between fluoxetine, gut microbiota alterations, and antidepressant treatment response.

Reference	Study Desing	Population/Model	Main Findings	Relevance to Antidepressant Efficacy Evidence	Relevance to SSRI-Associated Weight Gain
Zhang et al. [40]	Experimental animal study	Rat model of CUMS	Fluoxetine partially reversed stress-induced dysbiosis and altered metabolic pathways.	Preclinical mechanistic support for a role of microbiota function in antidepressant efficacy.	Not directly assessed
Ty et al. [45]	Experimental microbiological study	Bacterial strains exposed to sublethal antidepressant concentrations	Fluoxetine induced multidrug efflux pumps and bacterial cross-resistance to antibiotics	Not directly assessed	Not directly assessed
Sun et al. [43]	Experimental animal study	Mouse model of CUMS	Fluoxetine increased microbial diversity, reduced opportunistic bacteria, and restored microbial balance; normalization correlated with behavioral improvement.	Preclinical evidence supporting an association between microbiota restoration and antidepressant effects.	Not assessed
Fung et al. [41]	Experimental animal + bacterial study	Mice; *Turicibacter sanguinis*	Fluoxetine inhibited bacterial serotonin uptake and reduced *T. sanguinis* colonization.	Mechanistic evidence for direct microbiota modulation by fluoxetine; mediation of antidepressant efficacy not directly tested.	Not assessed
Lyte et al. [42]	Experimental animal study	Healthy mice	Fluoxetine selectively remodeled gut microbiota and was associated with differences in body weight.	Not directly assessed.	Preliminary evidence of an association with metabolic outcomes; mediation not established.
Lee et al. [44]	Experimental animal study	LPS-induced mouse model of depression	Microbiota depletion attenuated fluoxetine efficacy; selected *Lactobacillus* species partially reproduced antidepressant effects; vagotomy abolished effects.	Strongest preclinical evidence supporting a mediating role of gut microbiota in fluoxetine efficacy.	Not assessed.

**Table 4:** Main effects of SSRIs on the gut microbiome and their potential relevance to antidepressant efficacy.

Drug	Changes in Gut Microbiota Composition and Diversity	Microbial Functional Effects	Potential Relevance to Antidepressant Efficacy	Evidence Relevant to SSRI-Associated Weight Gain	Predominant Type of Evidence
Sertraline	Selective remodeling of the gut microbiota; ↓ α-diversity; alterations in the abundance of specific bacterial taxa	Antimicrobial activity; inhibition of biofilm formation; effects on membrane integrity, efflux pumps, and microbial metabolism	Direct microbiota modulation; possible contribution to antidepressant effects through gut–brain pathways	No direct evidence; contribution to SSRI-associated weight gain remains hypothetical	Predominantly in vitro and animal studies; limited clinical evidence
Escitalopram	Partial restoration of dysbiosis; ↓ Firmicutes/Bacteroidetes ratio; ↑ relative abundance of *Bacteroides*	Alterations in tryptophan-, phospholipid-, and sphingolipid-related pathways; associations with microbial metabolites including IPA	Microbiota composition and metabolic potential are associated with treatment response; microbial metabolites may be potential biomarkers	Not directly assessed	Clinical studies and animal models
Fluoxetine	↑ Microbial diversity; attenuation of dysbiosis; ↓ opportunistic bacteria; modulation of *Turicibacter* and *Lactobacillus* abundance	Modulation of amino acid, lipid, and carbohydrate metabolism; effects on vitamin biosynthesis and microbial metabolites; direct effects on selected bacterial species	Regulation of amino acid, lipid, and Preclinical evidence suggests a potential mediating role of microbiota in antidepressant efficacy, involving gut–brain/vagal signaling	Preliminary association between microbiota remodeling and body weight in mice; causal mediation not established	Predominantly animal studies; limited clinical evidence

↑ increase; ↓ decrease.

## Data Availability

No new data were created or analyzed in this study. Data sharing is not applicable to this article.

## Supplementary Materials

The following supporting information can be downloaded at: https://www.mdpi.com/article/10.3390/nu18182940/s1, Table S1: SANRA self-assessment of the narrative review.

## Abbreviations

The following abbreviations are used in this manuscript:

SSRIs	selective serotonin reuptake inhibitors
SNRIs	serotonin–norepinephrine reuptake inhibitors
SERT	serotonin transporter
NET	norepinephrine transporter
MeSH	medical Subject Headings
SCFA	short-chain fatty acid
ROS	reactive oxygen species
MDD	major depressive disorder
IPA	indole-3-propionic acid
RND	resistance–nodulation–division
CUMS	chronic unpredictable mild stress
LPS	lipopolysaccharide
IL-6	interleukin-6
IL-1β	interleukin-1β
TNF-α	tumor necrosis factor-α
CNS	central nervous system
GABA	γ-aminobutyric acid
BDNF	brain-derived neurotrophic factor
CFU	colony-forming unit
GPCRs	G protein-coupled receptors
GLP-1	glucagon-like peptide-1
PYY	peptide YY
HDACs	histone deacetylases
Tregs	regulatory T cells
BMI	body mass index
HOMA-IR	homeostasis model assessment of insulin resistance
FFAR2	free fatty acid receptor 2
IPE	inulin–propionate ester
POMC	proopiomelanocortin
AgRP	agouti-related peptide
ZO-1	zonula occludens-1
MAMPs	microbe-associated molecular patterns
NF-κB	nuclear factor kappa B NF-κB
AP-1	activator protein-1
JNK	c-Jun N-terminal kinase
SPMs	specialized pro-resolving mediators
IRS-1	insulin receptor substrate-1
PI3K/Akt	phosphoinositide 3-kinase/protein kinase B signaling pathway
AhR	aryl hydrocarbon receptor
MedDiet	Mediterranean diet
FOSs	fructooligosaccharides
GOSs	galactooligosaccharides
FMT	fecal microbiota transplantation
