# Supplementary material for: Weight Gain During SSRI Therapy: The Potential Role of the Gut Microbiota—A Narrative Review and Mechanistic Hypothesis
Source: Nutrients. 2026 Sep 8;18(18):2940. doi: 10.3390/nu18182940 (PMC13610818; doi:10.3390/nu18182940)
Supplement: Supplementary file 1 [file nutrients-18-02940-s001.zip › nutrients-4504545-supplementary.pdf]

Supplementary Table S1. SANRA self-assessment of the narrative review

No.	SANRA item	Score	Justification based on the manuscript
1	Justification of the article's importance for the readership	2	The Introduction explains the clinical relevance of SSRI-associated weight gain, its potential consequences for treatment adherence and health, and the unresolved role of the gut microbiota.
2	Statement of concrete aims or formulation of questions	2	A specific aim is stated at the end of the Introduction: to critically summarize evidence concerning SSRI therapy, gut microbiota, and body weight regulation and to propose an integrative mechanistic framework.
3	Description of the literature search	1	The Methods section provides information about the databases, search period, language restrictions, search subject areas, search terms, eligibility criteria, and evidence assessment criteria. However, there is no graphical representation of the study procedure.
4	Referencing	2	All key statements in the narrative sections and tables are supported by references.
5	Scientific reasoning	2	The manuscript distinguishes clinical, preclinical, and in vitro evidence, separates direct from indirect evidence, considers confounding and bidirectional relationships, and repeatedly identifies the proposed mechanisms as hypothetical rather than causal.
6	Appropriate presentation of data	1	Quantitative data are provided for the principal studies of SSRI-associated weight gain, including sample size, weight differences, incidence rates, and relative risk estimates. However, several mechanistic studies are presented only qualitatively, and the summary tables do not consistently report sample sizes, effect estimates, or measures of uncertainty.
	**Total score**	**10/12**	
